# BST-silicon hybrid terahertz meta-modulator for dual-stimuli-triggered opposite transmission amplitude control

**DOI:** 10.1515/nanoph-2022-0018

**Published:** 2022-03-17

**Authors:** Bowen Dong, Cheng Zhang, Guanxuan Guo, Xueqian Zhang, Yuchao Wang, Lingling Huang, Hua Ma, Qiang Cheng

**Affiliations:** Department of Basic Sciences, Air Force Engineering University, Xi’an, 710038, China; Hubei Engineering Research Center of RF-Microwave Technology and Application, School of Science, Wuhan University of Technology, Wuhan, 430070, China; Center for Terahertz waves and College of Precision Instrument, Optoelectronics Engineering and the Key Laboratory of Optoelectronics Information and Technology (Ministry of Education), Tianjin University, Tianjin, 300072, China; School of Optics and Photonics, Beijing Institute of Technology, Beijing, 100081, China; Department of Radio Engineering, State Key Laboratory of Millimeter Waves, Southeast University, Nanjing, 210096, China

**Keywords:** dual external stimuli, opposite transmission amplitude control, terahertz meta-modulator

## Abstract

With the drafting of the 6G white paper, terahertz (THz) modulators reshow profound significance in wireless communication, data storage, and imaging. Active tuning of THz waves through hybrid meta-structure incorporated with smart materials has attracted keen interest due to the deliberate structural design and dynamic transition of material properties. However, until now, these meta-devices have usually been responsive to a single driving field, such as electrical, thermal, or optical stimuli, which hinders their applicability for multidimensional manipulation of THz waves. Herein, to the best of our knowledge, a Ba_0.6_Sr_0.4_TiO_3_–silicon hybrid meta-modulator to achieve opposite tuning of the amplitude characteristic with two different types of stimuli is proposed for the first time. When driven by an external voltage, the proposed meta-modulator exhibits enhanced transmittance. In contrast, the transmission coefficient gradually decays as the external current increases. This outstanding performance is systematically studied by analyzing the carrier transport in the meta-structure as well as the change in the dielectric constant. Our research provides a novel idea for the development of actively tunable THz meta-devices and paves the way for robust multifunctionality in electrically controlled THz switching, and biosensors.

## 1 Introduction

Terahertz (THz) waves that carry vital physical information can be adopted in cutting-edge technologies such as high-speed wireless communications [1], [2], [3], [4], on-chip communications [5] biomedical diagnostics [6], [7], [8], security imaging [9], [10], [11], signal detection [12], and quality control [13], [14], [15]. In particular, the THz band is naturally a reliable choice for future 6G communication (from 275 to 450 GHz) because of its large communication capacity, good directivity, and robust anti-interference ability [3, 16, 17]. In a THz communication system, a modulator plays a key role in signal processing and can realize dynamic tuning of one or more parameters (amplitude and phase) of transmitted and/or reflected THz waves [18], [19], [20], [21]. Therefore, obtaining a THz modulator with stable performance, good modulation effect, and simple modulation mode has become a focus of current research.

Over the past decades, many efforts have been devoted to developing fast and efficient THz modulators by integrating metamaterials or metasurfaces with active materials (perovskites, GaAs and graphene) [22], [23], [24], [25], [26], [27], [28], which has driven the prompt development of the corresponding applications mentioned above. For example, an optically tuned THz meta-modulator consisting of an array of gold electric resonator elements fabricated on a GaAs substrate was proposed for flexibly controlling the amplitude of transmitted THz waves, the modulation depth of which could reach 50%, and the response speed could rise to 100 kHz [29]. With simultaneous optical and electrical excitations, Li et al. experimentally demonstrated an active diode for THz waves consisting of a graphene–silicon hybrid film. The proposed meta-diode provided a significant transmission modulation of 83% in the graphene–silicon hybrid film, which exhibited tremendous potential for applications in designing broadband THz modulators and switchable THz plasmonic and metamaterial devices [30]. Furthermore, Zhang et al. proposed an active and enhanced resonant metamaterial embedded with a nanostructured two-dimensional electron gas (2DEG) layer of a GaN high electron mobility transistor (HEMT), which could realize efficient phase modulation (greater than 150° in simulation) of THz waves [31]. In the dynamic experiments, a 137° phase shift was achieved with an external control voltage of only several volts in the THz transmission mode, providing a promising approach for developing a fast and effective phase modulator in THz application systems. Despite the rapid progress in the corresponding field, with regard to any specified meta-modulator, it can usually only be triggered by a single external excitation, such as an applied current or a thermal or mechanical field, which restricts its applications in some special scenarios. Some smart materials, such as vanadium dioxide (VO_2_), are simultaneously sensitive to dynamic changes in diverse physical fields [32], [33], [34], [35], which facilitates achievement of a multiphysical THz meta-modulator with the aid of rational structural design. However, the final states of the same materials after being driven by different stimuli are approximately identical, which confines the functional expansion of the existing meta-device. Therefore, obtaining a THz modulator that can realize different features when driven by different stimuli is urgently necessary.

**Figure 1:**
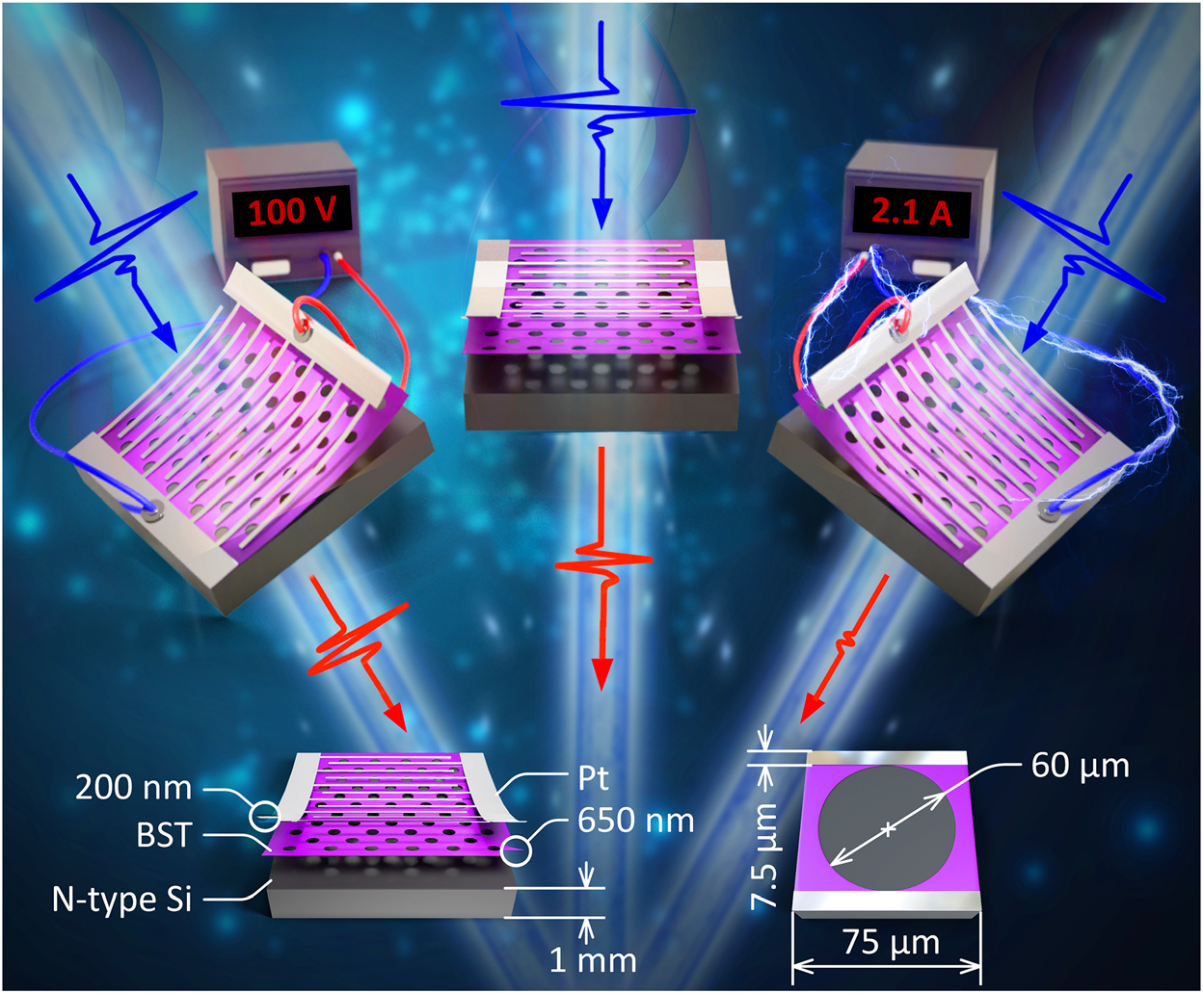
Schematic diagram of the proposed BST–silicon hybrid meta-modulator for tuning transmitted THz waves under different stimulus modes (left side for voltage tuning mode, right side for current tuning mode, and middle for comparison). Inset: detailed parameters of the proposed meta-atom.

To address the abovementioned problem, in this paper, we propose a Ba_0.6_Sr_0.4_TiO_3_ (BST)-silicon hybrid meta-modulator with two tuning modes that have opposite tuning effects. The proposed meta-modulator adopts a three-layer structure (Figure 1), the top layer of which contains an array of platinum (Pt) interdigital electrodes (with a thickness of 200 nm) that can provide tunable bias voltage and bias current for the proposed meta-atom. The BST film (with a thickness of 650 nm) etched with periodic circular holes is located in the middle of the entire structure. The bottom layer is a full-layered N-type silicon substrate with a thickness of 1.0 mm. Through the deliberate design, for the first time, two contrasting tuning modes (termed the voltage tuning mode and current tuning mode) are successfully realized with the proposed THz meta-modulator. Under the voltage tuning mode, the transmittance of THz waves (the E-field direction of which is parallel to the Pt electrodes) can be enhanced as the voltage increases. In contrast, with increasing external current, the current tuning method can restrain the transmitted THz signals until they are nearly shielded. In addition, at thresholds of 100 V (on the left side of Figure 1) and 2.1 A (on the right side of Figure 1), the modulation depth of our design can reach 29.4 and 88.2%, respectively. These unique features could enable novel devices for efficient and flexible manipulation of THz waves with regard to complex external excitations.

## 2 Results and discussion

### 2.1 Tuning mode and performance

**Figure 2:**
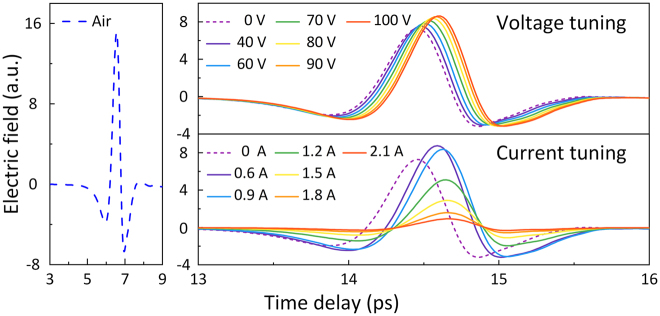
Time-domain transmittance signals of the proposed dual-mode meta-modulator and their amplitude change under different external voltage and current excitations. The signal waveform of the reference (blue dotted line) is plotted on the left side.

Figure 2 shows the experimental THz time-domain spectra of the proposed meta-atom (Figure 1) under varying external voltages (top right corner of Figure 2) and currents (bottom right corner of Figure 2). A blank sample (air) is also provided in Figure 2 as a reference for subsequently calculating the frequency-domain transmittance coefficient. All the corresponding results are measured through a THz time-domain spectroscopy (THz-TDS) system, and notably, when conducting the experiments, only the time-domain signals from 0 to 20 ps are collected to avoid the multipath effect and eliminate Fabry–Perot reflection, which can directly affect the test precision even cause obvious errors. The signal waveforms transmitted through the fabricated sample show totally different changes with increasing current and voltage amplitude. With regard to the voltage tuning mode, the amplitude of the time-domain signal increases from 7.27 ps @ 0 V to 8.63 ps @ 100 V and the time-domain peak shifts from 14.45 ps @ 0 V to 14.6 ps @ 100 V as the input voltage gradually increases. Furthermore, once the applied electric current is boosted to above 0.6 A, the transmitted THz main pulse rapidly decreases, while little pulse position shift can be observed. From the time-domain signal waveforms shown in Figure 2, the basic functionality and operation method of the proposed hybrid meta-atom can be qualitatively revealed to be as desired, but the performance parameters of the dual-mode modulation, such as the modulation depth, cannot be intuitively exhibited. Hence, the frequency-domain spectra of the transmitted THz waves need to be provided, which contain more information such as amplitude and phase.

**Figure 3:**
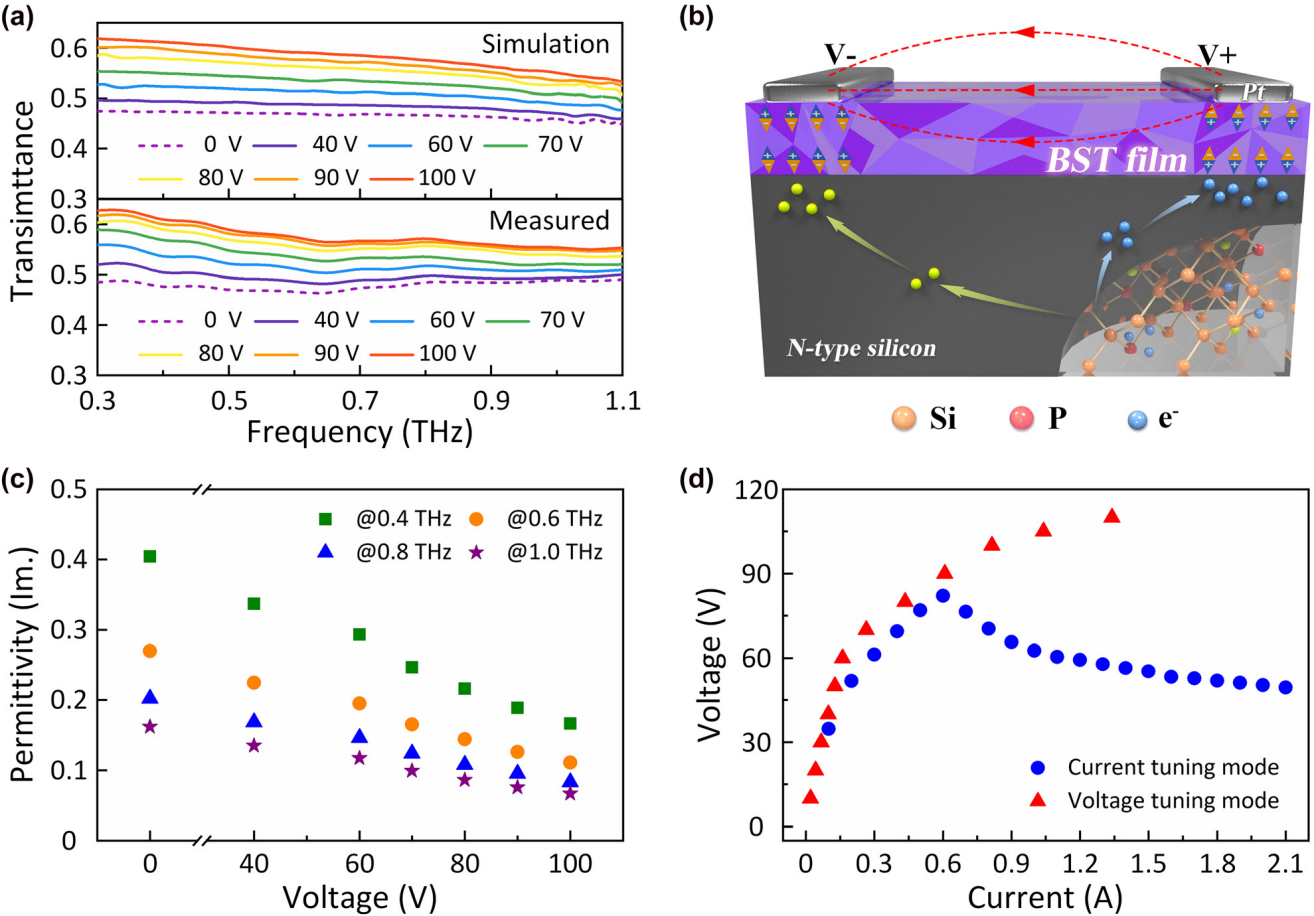
Voltage tuning mode (a) Simulated and measured transmittance of the hybrid meta-modulator under different bias voltages. (b) Operating schematic illustration of the meta-modulator under the voltage tuning mode. (c) Voltage dependence of the imaginary part of the effective permittivity for our design at different frequencies. (d) Measured voltage–current relation of our design under current tuning mode and voltage tuning mode.

**Figure 4:**
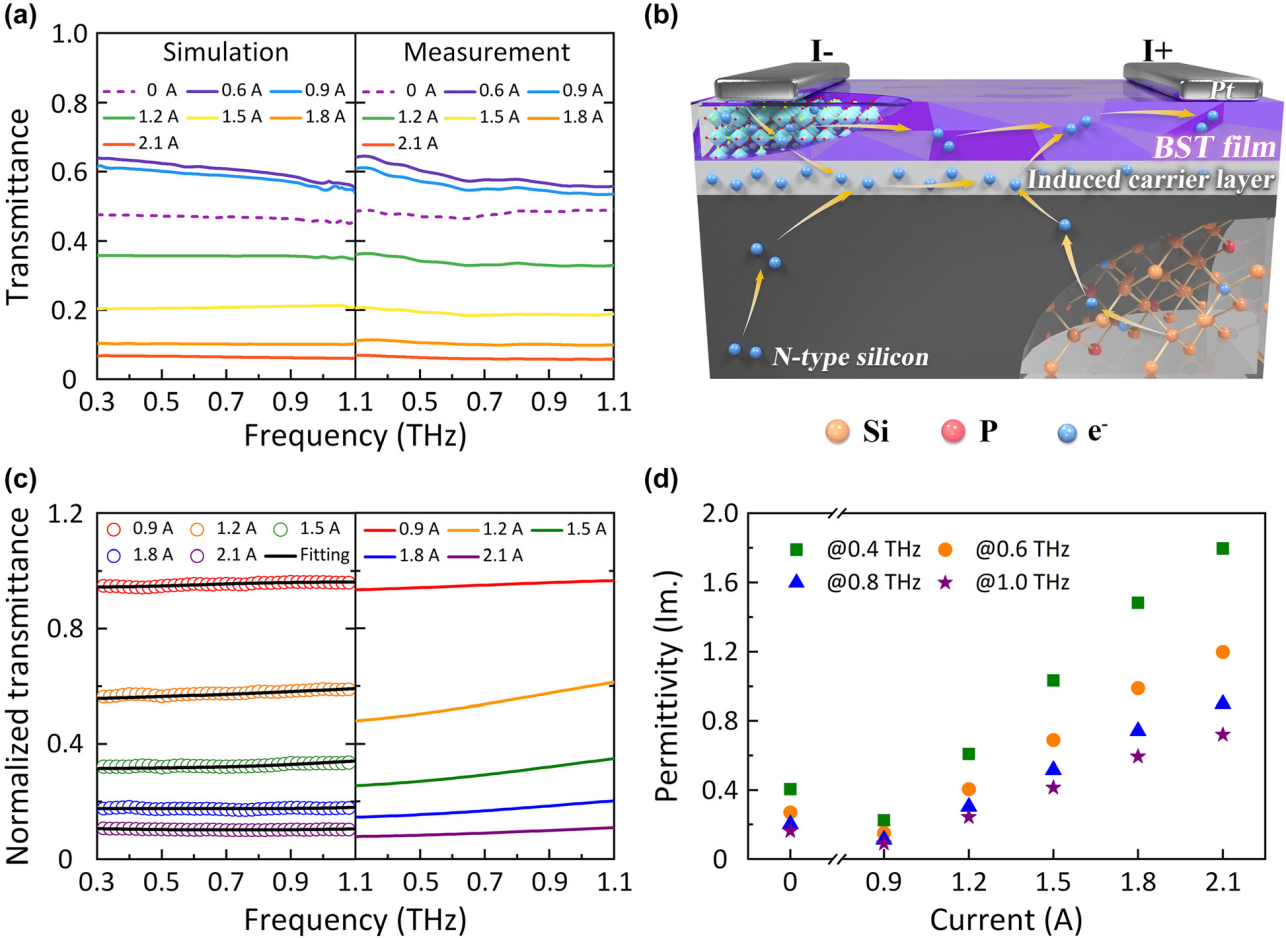
Current tuning mode (a) Simulated and measured transmittance of the hybrid meta-modulator under different currents. (b) Operating schematic diagram of the meta-modulator under the current tuning mode. (c) Measured (circle), fitted (solid black curve) and theoretical (colored solid curve) values of the normalized transmission amplitude as a function of frequency at bias currents of 0.9, 1.2, 1.5, 1.8, and 2.1 A. (d) Current intensity dependence of the imaginary part of the effective permittivity for our design at different frequencies.

By performing a fast Fourier transformation (FFT) of the experimental data in Figure 2, the frequency-dependent amplitude (T(ω)) of the THz pulse transmitted through the hybrid metamaterial can be obtained, as shown in Figures 3a and 4a. Here, free space (blank sample) is considered as the reference, which can be used to normalize the electric field intensity as follows:

(1) $$\left|T\left(\omega \right)\right|=\left|\frac{E_{\text{Sample}}\left(\omega \right)}{E_{\text{Ref}\text{.}}\left(\omega \right)}\right|,$$

in which $E_{\text{Sample/Ref}\text{.}}\left(\omega \right)$ is the *E*-field calculated from the time-domain data of the sample/reference, as illustrated in Figure 2. Figures 3a and 4a show the calculated transmission coefficient through the BST–silicon hybrid meta-modulator with different applied currents and voltages in the frequency range of 0.3 THz to 1.1 THz.

From Figure 3a, the all-band (from 0.3 THz to 1.1 THz) transmission of THz waves through the sample is obviously gradually enhanced as the input voltage applied on the Pt interdigital electrodes increases from 0 to 100 V, which is consistent with the measured time-domain results (Figure 2). In addition, the opposite phenomena can be observed in Figure 4a. Once a DC current flow greater than 0.6 A is brought into the amplitude modulation system along the Pt interdigital electrodes, the transmittance rate rapidly attenuates, as predicted in Figure 2.

To further demonstrate the dynamic modulation ability of the proposed meta-device, the amplitude modulation depth is defined as

(2) $$M_{\text{V}}\left(\omega \right)=\left|\frac{\left(T_{V_{\text{g}}}\left(\omega \right)-T_{V_{0}}\left(\omega \right)\right)}{T_{V_{0}}\left(\omega \right)}\right|,$$

where $T_{V_{\text{g}}}\left(\omega \right)$ and $T_{V_{0}}\left(\omega \right)$ are the normalized transmission at ω in terms of *V*_0_ (0 V) and *V*_g_ voltages, respectively. According to Eq. (2), the calculated amplitude modulation depth reaches an average value of 20.5% from 0.3 THz to 1.1 THz when the bias voltage is equal to 100 V. Similarly, the amplitude modulation depth under various applied currents can be calculated as

(3) $$M_{\text{I}}\left(\omega \right)=\left|\frac{\left(T_{I_{\text{g}}}\left(\omega \right)-T_{I_{0}}\left(\omega \right)\right)}{T_{I_{0}}\left(\omega \right)}\right|,$$

in which $T_{I_{\text{g}}}\left(\omega \right)$ and $T_{I_{0}}\left(\omega \right)$ are the normalized transmission rates for 0 and *I*_g_ currents at ω . When the input current rises to 2.1 A, the modulation depth is greater than 85.8% during the operation bandwidth, making our design an excellent THz switch. Until now, we have introduced the tuning method and the corresponding modulation performance of the proposed hybrid metamaterial in detail. In the next section, we will deeply investigate the physical mechanism of the interesting characteristics observed above.

### 2.2 Physical mechanism

In our design, N-type silicon with abundant free electrons is chosen as the bottom substrate, the inside carrier distribution of which can be flexibly adjusted under various external stimuli, thus making it convenient to influence the transmittance features of the incident THz waves [36]. To confine the flow of the electric charges when driven by voltage or current fields, the BST film is patterned as a circular-hole array, and the Pt electrodes are arranged on both sides [37]. Note that due to the inevitable generation of oxygen vacancies during the coating process, the BST thin film that has few free charges can be regarded as a high-resistance semiconductor.

With regard to the voltage tuning mode, to reveal its physical mechanism, the microscopic behavior of the carriers inside the meta-atom is provided in Figure 3b. An interdigital electrode is used to apply a potential difference between the adjacent electrodes. Once an external voltage field is introduced, the patterned BST layer can be polarized along the electric field line from the anode to the cathode (Figure 3b). Due to the special hole-shaped design (Figure 1) that removes the redundant BST in the central part, the induced polarization phenomena can clearly be found to mostly occur below the Pt electrodes. Near the anode, the induced electric field in the BST layer is vertical to the surface and points to the positive pole, while the opposite electric field line can be achieved below the cathode. Hence, owing to the polarized BST, an opposite potential distribution to that of the input voltage field is produced at the boundary between BST and N-type silicon, which can directly determine the behavior of the carriers inside the bottom silicon. As shown in Figure 3b, under the influence of the extra field caused by BST polarization, some of the disordered free charges and holes in the silicon can be separated and driven to the corresponding regions (Figure 3b) by the electric force, resulting in a decrease in the conductivity. Therefore, the dielectric loss of the entire meta-structure gradually decays with the regression of the dielectric nature of the adopted N-type silicon, and accordingly, the THz transmittance is improved. In the following, to uncover the effect of the dynamic bias voltage on the modulation depth, the induced electrostatic potential difference (*V*_EP_) is calculated as [37]

(4) $$V_{\text{EP}}=\frac{SP}{C},$$

where *C* is the capacitance between the electrodes with an area of *S*. *P* determined by the external electric voltage denotes the polarization degree of BST. In Eq. (4), *S* is fixed once the meta-device is fabricated. Additionally, we consider *C* to be a constant parameter for the thin layer BST patterns that has little effect on the capacitance between the electrodes when various external voltages are applied. Therefore, only *P* can be used to adjust the induced electrostatic potential difference formed at the interface between the BST-patterned layer and the N-type silicon substrate. That is, with increasing input electric voltage, the polarization degree of BST and induced *V*_EP_ can be synchronously promoted. Combined with the previous analysis, more carriers in the silicon can be confined beneath the corresponding electrodes, and their dissipation of the incident THz waves can be gradually alleviated, thereby leading to enhancement of the transmitted power. Based on this theoretical model (Figure 3b), the simulated results also have been revealed in Figure 3a, showing great consistent with the measured ones (see Supplementary Note S1 for a detailed introduction of the simulation method).

To further reveal the voltage tuning mechanism, the imaginary part of the effective dielectric constant of the meta-modulator is extracted from the THz transmittance spectra (Figure 2). Based on the classical model proposed by L. Duvillaret and T. D. Dorney [38], [39], [40], the complex refractive index of the total structure can be expressed as

(5) $$\tilde{n}=n\left(\omega \right)-j\kappa \left(\omega \right),$$

in which n(ω) represents the real refractive index and κ(ω) denotes the extinction coefficient, which describes the absorption characteristics of the sample. Furthermore, according to the interaction between the sample and THz waves, the transmitted signal can be described as

(6) $$T\left(\omega \right)=\frac{4\tilde{n}{\text{e}}^{\frac{j2\pi \left(\tilde{n}-1\right)\omega d}{c}}}{{\left(1+\tilde{n}\right)}^{2}}=A\left(\omega \right){\text{e}}^{j\varphi \left(\omega \right)},$$

where A(ω) and φ(ω) are the amplitude and phase of the transmitted signal, respectively, *d* is the thickness of the sample, and *c* is the speed of light in vacuum. Therefore, by substituting Eq. (5) into Eq. (6), n(ω) and κ(ω) can be derived as follows:

(7) $$n\left(\omega \right)=\frac{\varphi \left(\omega \right)c}{\omega d}+1\text{ and}$$

(8) $$\kappa \left(\omega \right)=\frac{c}{d\omega }\text{ln}\frac{4n\left(\omega \right)}{A\left(\omega \right){\left(n\left(\omega \right)+1\right)}^{2}}.$$

Finally, since the complex permittivity has relationships with n(ω) and κ(ω) of

(9) $$\varepsilon_{\text{r}}=n{\left(\omega \right)}^{2}-\kappa {\left(\omega \right)}^{2}\text{ and}$$

(10) $$\varepsilon_{\text{i}}=2n\left(\omega \right)\kappa \left(\omega \right),$$

the real part $\left(\varepsilon_{\text{r}}\right)$ and imaginary part $\left(\varepsilon_{\text{i}}\right)$ of the effective permittivity can be obtained by substituting Eqs. (7) and (8) into Eq. (9) and (10), respectively. In Figure 3c, the calculated $\varepsilon_{\text{i}}$ corresponding to different bias voltages is illustrated, and the dielectric loss of the entire structure clearly gradually decreases as the input voltage increases, which agrees well with the above qualitative analysis.

Thus far, we have comprehensively revealed the inner physical mechanism of the proposed meta-modulator under the voltage tuning mode. Next, we will perform an in-depth investigation of the physical source of the current tuning mode.

With regard to the current tuning mode, two different processes successively impact the transmission characteristics of the proposed meta-modulator. In the first process, as the bias current increases to 0.6 A from the initial condition (0 A), a similar phenomenon to that in the voltage tuning mode can be observed by comparing the transmittance spectra in Figure 4a with those in Figure 3a. Generally, a current source can provide a stable output current without considering the voltage between the electrodes. Hence, due to the high resistance property of the BST film, a relatively high voltage can be generated by the current supply even when the bias current is small, resulting in almost the same modulation effect as the voltage tuning mode. In addition, this phenomenon also can be revealed from the overlapped voltage–current curves (Figure 3d), which denote the identical physical process.

In the second process, as the applied current further increases (exceeding 0.6 A), free electrons can be injected into the boundary between the patterned BST film and the silicon layer across the hole structures etched in the BST film, leading to the generation of a thin carrier layer at the corresponding locations (Figure 4b). In the meantime, for the conductive path is successfully built, the load resistance inner our design significantly decreases, resulting in the reduction of the input voltage as the bias current is more than 0.6 A (Figure 3d).

Therefore, as the bias current increases from 0.6 to 2.1 A, the carrier concentration of the assumed conducting layer significantly increases as desired, resulting in dissipation of the incident THz power. To verify this hypothesis, we study the effect of the induced carrier layer and its carrier density on THz transmittance. Since the induced carrier layer is quite thin [41], a theoretical model is chosen to obtain the approximate transmittance |T(ω)| of the total structure as follows [30, 41], [42], [43]:

(11) $$\left|T\left(\omega \right)\right|=\left|\frac{1}{1+NZ_{0}\frac{\sigma \left(\omega \right)}{\left(1+n_{\text{sub}}\right)}}\right|,$$

where *N* refers to the equivalent layer number of the induced carrier layer, *Z*_0_ is the vacuum impedance, and *n*_sub_ is the effective refractive index of the proposed meta-modulator at 0.6 A. Note that all the measured, fitted, and theoretical transmittances shown in Figure 4c are normalized to the condition in which the bias current is equal to 0.6 A. In addition, σ(ω) in Eq. (11) is the complex conductivity of the induced carrier layer, which can be approximately described by the Drude model [41]:

(12) $$\sigma \left(\omega \right)=\frac{ne^{2}}{m^{*}}\times \frac{1}{\frac{1}{\tau }-i\omega },$$

in which *n* is the carrier density of the induced carrier layer, and τ is the carrier relaxation time. *e* and m* are the charge and effective mass of an electron. Therefore, the normalized transmittance can be calculated by substituting Eq. (12) into Eq. (11). Furthermore, once the structure and the adopted materials are decided, *N*, *Z*_0_, *n*_sub_, *e*, m*, and τ are fixed, and thus, only the parameter *n* can be optimized to force the calculated |T(ω)| approach to the fitted value (black lines in the left part of Figure 4c). After several rounds of optimization, the carrier density is confirmed to be 1.1 × 10^17^ cm^−3^ @ 0.9 A, 15 × 10^17^ cm^−3^ @ 1.2 A, 41 × 10^17^ cm^−3^ @ 1.5 A, 81 × 10^17^ cm^−3^ @ 1.8 A and 160 × 10^17^ cm^−3^ @ 2.1 A. As previously predicted, the fitted carrier density is positively associated with the applied current. The final calculated results of the normalized transmittance are illustrated in the right part of Figure 4c, which are consistent with the measured and fitted results in the left part, proving the correctness of our previous hypothesis. At the same time, we conduct the simulation of the proposed theoretical model (Figure 4b) by utilizing CST Microwave studio, and the simulated results demonstrate excellent agreement with the measured ones (Figure 4a), further verifying this analytical method (see Supplementary Note S1 for a detailed introduction of the simulation method).

Additionally, the dielectric loss of the entire meta-structure under different bias currents is calculated based on Eqs. (7)–(10) and provided in Figure 4d. When the input current increases, more incident power can clearly be dissipated by our design as a result of the promotion of the imaginary part of the permittivity (Figure 4d) of the entire structure, which further reveals the current tuning mechanism from the aspect of the equivalent medium.

## 3 Conclusions

In summary, we experimentally verified a hybrid meta-modulator that can adjust the THz transmission amplitude with opposite effects when excited by different external stimuli. In the voltage tuning mode, once the patterned BST film is polarized, the carriers in the silicon can be redistributed with the help of the induced potential difference, which brings about a decline in the dielectric loss of the entire structure as the free electrons and holes are tightly bound near the Pt electrodes. In comparison, in the current tuning mode, an induced carrier layer is formed at the interface between the patterned BST layer and the N-type silicon substrate with the injection of external electrons by a current supply. The carrier concentration of the conducting layer is enhanced as the applied current increases, thus gradually suppressing the transmittance of the incident THz waves. In addition, the influences of the carrier distribution and carrier density on incident THz waves are all proven by theoretical models and approximate calculations, which are in good agreement with the corresponding assumptions. We believe that our proposed meta-modulator represents a remarkable advancement toward metasurfaces/metamaterials enabling multidimensional responses and multifunctionalities, and this novel design method, which is compatible with traditional silicon-based technology, can be extended to infrared (IR) spectra and even optical bands for wider applications.

## 4 Experimental section

### 4.1 Coating process

To fabricate the proposed meta-device, a three-layer substrate (Pt–BST–silicon) is first prepared for the subsequent lithography process. During the coating process, the BST film with the optimized thickness is primarily deposited on a clean N-type silicon wafer through radio frequency (RF) magnetron sputtering with a deposition speed of approximately 50 nm/h. Then, a 200 nm thick Pt film is deposited on the semi-finished sample by direct current (DC) magnetron sputtering with a sputtering rate of approximately 10 nm/min. Notably, the prepared BST film needs to be annealed in a 650 °C pure oxygen atmosphere to facilitate growth of the lattice and adequate filling of the oxygen holes.

### 4.2 Lithography process

Before conducting overlay lithography, the photoresist is evenly coated on the top surface of the manufactured three-layer substrate through a two-step method with the help of a spin coater (Laurell EDC-650Mz-23NPPB). Then, the sample is in turn treated with soft baking (at 100 °C for 60 s) and hard baking (after exposure) at 110 °C for 90 s to evaporate the residual solvent and release the extra stress in the photoresist film. In addition, these annealing treatments can help improve the adhesion between the photoresist and the sample and reduce the standing wave effect.

**Figure 5:**
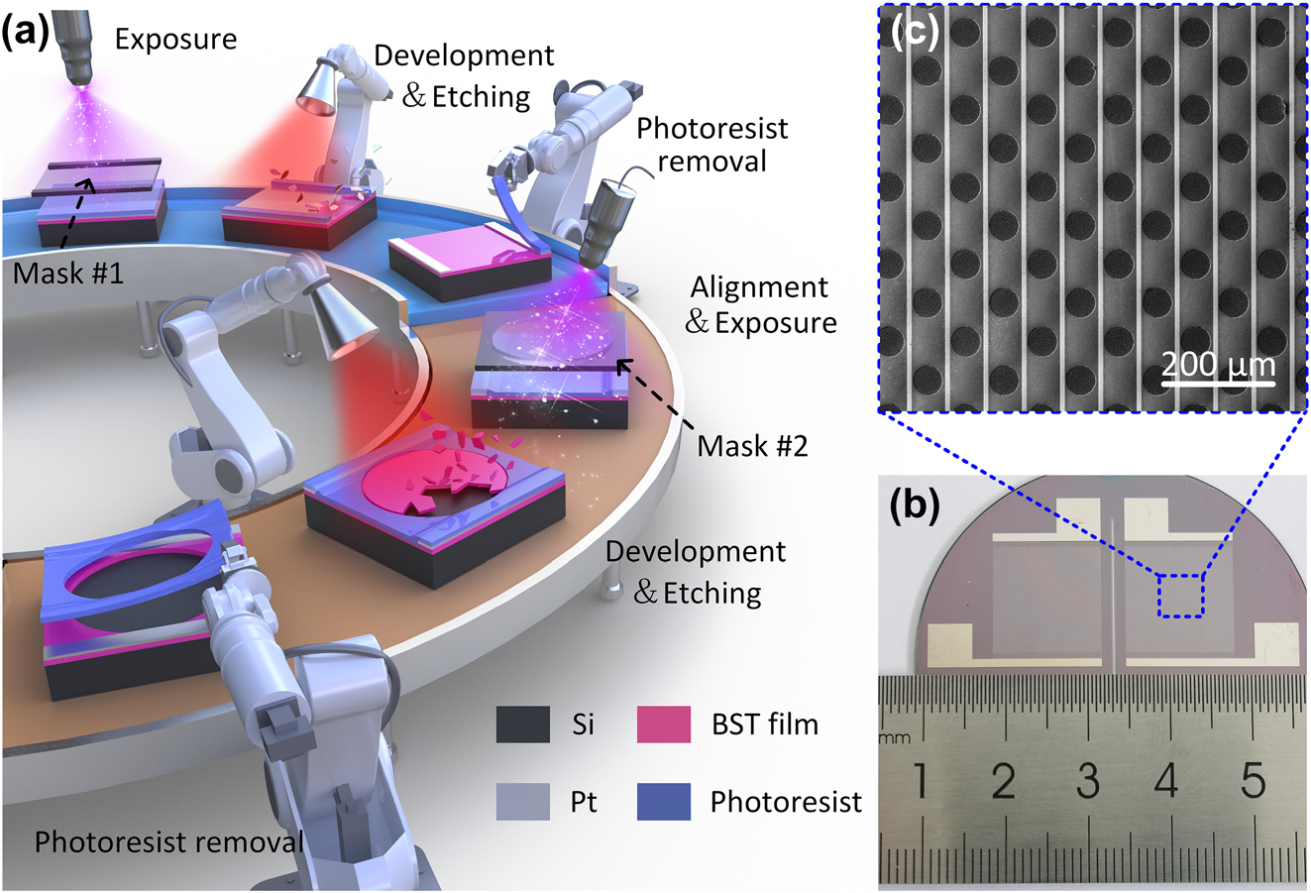
Fabrication process and sample display (a) Schematic diagram of the overlay lithography process. Photograph (b) and SEM image (c) of the fabricated BST–silicon hybrid meta-modulator.

In the following, traditional semiconductor technology, as shown in Figure 5a, is adopted to fabricate the overlapping patterns. First, mask #1 for manufacturing Pt interdigital electrodes is utilized for the exposure process, which can transfer the predesigned complementary pattern to the photoresist. Then, the photosensitive region can be removed through chemical development and ion beam etching while the other part is protected and wiped away in the photoresist removal step. The fabrication process mentioned above can be observed in the blue part of Figure 5a. Next, with the help of mask #2, periodic circular holes are etched in the middle-layer BST film by utilizing a similar preparation method as before. Note that overlay technology is the key point during the second round of the photoetching process, determining the quality of the final sample. Here, to overcome this challenge, we have left several alignment marks at identical locations (always along the diagonal line) of both mask #1 and mask #2 in advance. When the marks remaining in the preliminary sample are matched with those in mask #2, the overlay operation can be finished with high precision. The procedure of the overlay lithography is provided in the brown part of Figure 5a.

As shown in Figure 5b, ports (L-shaped structures) for connection to the external voltage and current sources are reserved around the array in the fabrication process. In addition, a scanning electron microscopy (SEM) image (Figure 5c) is also given to exhibit the excellent microscopic morphology of the sample, proving the high accuracy of the adopted overlay lithography.

## Supplementary Material

Supplementary Material

Supplementary Material
