# Supplementary material for: BST-silicon hybrid terahertz meta-modulator for dual-stimuli-triggered opposite transmission amplitude control
Source: Nanophotonics. 2022 Mar 17;11(9):2075–83. doi: 10.1515/nanoph-2022-0018 (PMC11501895; doi:10.1515/nanoph-2022-0018)
Supplement: Supplementary file 1 — Supplementary Material [file j_nanoph-2022-0018_suppl_001.docx]

Supplementary Information for

**BST-Silicon Hybrid Terahertz Meta-Modulator for Dual-Stimuli-Triggered Opposite Transmission Amplitude Control**

Bowen Dong^1,&^, Cheng Zhang^2,&,^*, Guanxuan Guo^3^, Xueqian Zhang^3^, Yuchao Wang^2^, Lingling Huang^4^, Hua Ma^1,^*, Qiang Cheng^5,^*

^1^Department of Basic Sciences, Air Force Engineering University, Xi’an, 710038, China

^2^Hubei Engineering Research Center of RF-Microwave Technology and Application, School of Science, Wuhan University of Technology, Wuhan, 430070, China

^3^Center for Terahertz waves and College of Precision Instrument, Optoelectronics Engineering and the Key Laboratory of Optoelectronics Information and Technology (Ministry of Education), Tianjin University, Tianjin, 300072, China

^4^School of Optics and Photonics, Beijing Institute of Technology, Beijing, 100081, China

^5^Department of Radio Engineering, State Key Laboratory of Millimeter Waves, Southeast University, Nanjing, 210096, China

^&^B. Dong, and C. Zhang contributed equally to this work.

*Correspondence and requests for materials should be addressed to C. Zhang (email: [czhang2020@whut.edu.cn](mailto:czhang2020@whut.edu.cn)), H. Ma ([mahuar@xjtu.edu.cn](mailto:mahuar@xjtu.edu.cn)) or Q. Cheng (email: qiangcheng@seu.edu.cn).

**This file includes:**

Supplementary Notes S1

**Supplementary Note S1: Detailed introduction of the simulation method**

The proposed meta-modular is built in CST Microwave Studio 2019 and placed in the unit cell boundary condition according to the optimized structural parameters. Floquet port with TE and TM modes, representing the *y*- and *x*-polarizations, respectively, are set as the excitation source.

Before conducting the simulation, a multilayer structure composed of the platinum electrode, BST, and silicon layers is first established. The material characteristics of lossy platinum are selected from the CST material library. The material characteristics of the BST layer are obtained through extracting parameters from the THz time-domain spectrum measurement data, and the relative dielectric constant of N-type silicon is set to 11.9.

**Regarding the voltage tuning mode**, according to the theoretical hypothesis in the main text, the modulation effect is mainly caused by the change of carrier distribution and density of silicon substrate. The disordered free charges and holes in the silicon can be separated and driven to the corresponding regions beneath the Pt electrodes by the electric force, resulting in a decrease in the conductivity and dielectric loss (our theoretical hypothesis). In the simulation process, THz transmission curves at different voltages can be simulated by changing the electric conductivity of the silicon layer. Therefore, to obtain the simulated results shown in Fig. 3(a) of the main text, we optimize the electric conductivity of the silicon layer according to the measured results at different voltages, and the high agreement also proves the correctness of our theoretical analysis.

**Regarding the current tuning mode**, the induced carrier layer is added at the interface between silicon and BST in the three-layer structure mentioned above, guided by the proposed theoretical hypothesis. As the current source continuously injects free electrons into the hybrid meta-modulator, the conductivity of the induced carrier layer increases with the increase of the input current, thereby blocking the transmission of the terahertz waves. We consider the uniformly distributed carrier layer as part of the silicon layer (at its top) in the simulation process. Then, by tuning the conductivity of the carrier layer and its thickness, the simulated results (Fig. 4(a) of the main text) as a function of input current can be acquired.
