# Supplementary material for: BST-silicon hybrid terahertz meta-modulator for dual-stimuli-triggered opposite transmission amplitude control
Source: Nanophotonics. 2022 Mar 17;11(9):2075–83. doi: 10.1515/nanoph-2022-0018 (PMC11501895; doi:10.1515/nanoph-2022-0018)
Supplement: Supplementary file 2 — Supplementary Material [file j_nanoph-2022-0018_suppl_002.pdf]

ACS Authoring Services

Editing Certificate

This document certifies that the manuscript

BST-Silicon Hybrid Terahertz Meta-Modulator for Dual-Stimuli-Triggered Opposite Transmission Amplitude Control

prepared by the authors

Bowen Dong, Cheng Zhang, Guanxuan Guo, Xueqian Zhang, Yuchao Wang, Yidan Su, Lingling Huang, Hua Ma, Qiang Cheng

was edited for proper English language, grammar, punctuation, spelling, and overall style by one or more of the highly qualified native English speaking editors at ACS.

This certificate was issued on **October 12, 2021** and may be verified on the ACS website using the verification code **FA4E-6997-ED57-BF01-059F** .

Neither the research content nor the authors' intentions were altered in any way during the editing process. Documents receiving this certification should be English-ready for publication; however, the author has the ability to accept or reject our suggestions and changes. To verify the final ACS edited version, please visit our verification page at secure.authoringservices.acs.org/certificate/verify.
If you have any questions or concerns about this edited document, please visit ACS support.

ACS provides a range of editing, translation, and manuscript services for researchers and publishers around the world.
For more information about our company, services, and partner discounts, please visit authoringservices.acs.org.
